# Severe malaria burden, clinical spectrum and outcomes at Apac district hospital, Uganda: a retrospective study of routine health facility-based data

**DOI:** 10.1186/s12936-023-04761-6

**Published:** 2023-10-25

**Authors:** Emmanuel Ocen, Ronald Opito, Crispus Tegu, Alex Oula, Peter Olupot-Olupot

**Affiliations:** 1 Department of Public Health, Faculty of Health Sciences, Busitema University, Mbale, Uganda; 2 Department of Health, Apac District Local Government, Apac, Uganda; 3 Departmemnt of Public Health, School of Health Sciences, Soroti University, Soroti, Uganda; 4 Department of Paediatric and Child Health, Faculty of Health Sciences, Busitema University, Mbale, Uganda; 5 Mbale Clinical Research Institute, Mbale, Uganda

**Keywords:** Child, Malaria, Hospitalization, Survivors

## Abstract

**Background:**

Most data describing severe malaria (SM) in sub-Saharan Africa (SSA) are from research settings outside disease endemic areas. Using routinely collected data from Apac District Hospital, this study aimed at determining the burden and clinical spectrum of severe malaria.

**Methods:**

This was a retrospective study that reviewed all paediatric admission records for malaria in the 24 months period from Jan 2019 to Dec 2020 at Apac District Hospital. Data on children aged 60 days to 12 years who at admission tested positive for malaria and fulfilled the World Health Organization clinical criteria for surveillance of severe malaria were abstracted using a customized proforma designed to capture variables on social demographic, clinical presentation, treatment, and outcomes. In addition, the tool included laboratory variables for complete blood counts, haemoglobin, and glucose levels. Data were analysed using STATA V15.0. The study had ethical approval from Mbale Regional Referral Hospital REC, Approval No. MRRH-REC 053/2019.

**Results:**

A total of 5631 admission records were retrieved for this study period. Of these, 3649 (64.8%) were malaria admissions and 3422/3649 were children below 12years, with only 1864 (54.5%) of children having complete data. Of the 1864 children, 745 (40.0%) fulfilled the severe malaria inclusion criteria. Of the 745 children, 51.4% (n = 381) were males. The median age at admission was 31 months (IQR = 17–60). The most common clinical presentations among children with severe malaria were fever 722 (97.3%), cough 478 (64.2%), and difficulty in breathing 122 (17.9%). The median length of hospital stay was 2 (IQR; 2–4) days and 133 (17.9%) had prolonged hospital stay (> 4 days). Factors independently associated with prolonged hospital stay were, presenting with difficulty in breathing, aOR 1.83 (95% CI 1.02–3.27, *P =* 0.042) and prostration aOR 8.47 (95% CI 1.94–36.99, *P =* 0.004). A majority of admitted children, 735 (98.7%) survived, while 10 (1.3%) died of SM.

**Conclusion:**

A high proportion (40.0%) of malaria admissions were due to SM. Prolonged Hospital stay was associated with prostration and difficulty in breathing. Overall mortality was low, 1.3% compared to mortality in the previously reported series. This study was able to use routinely collected data to describe the burden and clinical spectrum of SM. Improvement in the quality of data from such settings would improve disease descriptions for policy, monitoring of epidemics, response to interventions and to inform research.

## Background

Globally morbidity and mortality due to falciparum malaria remains high. In the pre-COVID-19 era, there were an estimated 229 million malaria cases in 2019 in 87 malaria endemic countries, declining from 238 million in 2000 [1]. In the post-COVID-19 era, sub-Saharan Africa (SSA) contributed 234 million malaria cases accounting for 95% of all malaria cases globally in 2021, with only four countries in the subcontinent contributing to almost 50% of the global burden in the same year, that is; Nigeria (27%), the Democratic Republic of the Congo (12%), Uganda (5%), and Mozambique (4%) [2]. The increase in malaria cases between 2019 and 2021 is attributed to the disruption of malaria prevention activities due to COVID-19 [2]. The most frequent cause of severe forms of malaria associated with poor outcomes is *Plasmodium falciparum* [3, 4]. The onset of symptoms of malaria infection follows a 7 to 14 day incubation period when the parasites undergo various developmental changes in the liver and blood [5]. In endemic areas, acquired partial immunity may modify the disease progression and outcomes [4]. In-patient mortality among children admitted with severe and complicated malaria varies across different epidemiological settings. Rural and low socio economic settings have been specifically linked to higher mortality compared with urban settings [6]. In Uganda, malaria is the 3rd most common killer in the general population, only surpassed by neonatal disorders and HIV/AIDS [7]. The situation seems worse in rural and remote places where poor access to health services affects the morbidity and mortality outcomes of the disease [1].

Current descriptions of SM are predominantly, if not, exclusively from few settings located in low transmission sites and mainly driven by research funding [8, 9]. Nonetheless the epidemiology of malaria differs with endemicity, burden and location. Moreover, outcomes due to malaria are contingent on age of patient, season of the year and transmission intensity [8, 10, 11]. The feasible way and means to ensure continuous surveillance of SM is through routine facility-based surveillance. These data if collected well have potential to play a key role in sustainability of SM surveillance. In addition, when systematically done, routine data have a huge role in updating the epidemiology on the disease in various geographical settings and over time. Furthermore, they have an untapped role of being a more direct route for providing feedback to the National Malaria Control Programme (NMCP) on the effectiveness of national interventions being implemented. Moreover, exploring routine facility-based data is critical for contributing data from non-research settings for a complete picture of disease. However, the potential of routinely collected data remains unexploited. The objective of this study was to determine the burden and clinical spectrum of severe malaria from the routine health facility-based surveillance at Apac District Hospital.

## Methods

### Study design and settings

This study was a retrospective study for admission records on children aged 2 months to 12 years admitted at Apac district hospital for the period Jan 2019 to Dec 2020. The hospital is located North of Lake Kyoga, 268 km North of Uganda’s Capital city, Kampala and 60 km west of Lira City. About 40% of the district is a marsh land covered with wetlands and swamps, which are conducive for mosquito breeding. The district has a total population of 368,626 people, of which 47.9% (176,571) are children less than 15 years of age [12]. The hospital has a bed capacity of 100 for in-patient services. In addition, it provides outpatient and basic laboratory services. Most of the severe malaria cases and complications are treated in the hospital, except in some cases where patients require specialized services for the disease complications such as severe renal failure requiring dialysis.

### Inclusion and exclusion criteria

All records for children aged 2 months to 12 years admitted with a confirmed diagnosis of malaria were included if they met any one or more of the World Health Organization (WHO) 2015 clinical criteria for surveillance of SM [13]. These criteria included severe anaemia (Hb less than 5 g/dL), prostration (generalized weakness so that the person is unable to sit, stand or walk without assistance), shock (compensated or decompensated), multiple convulsions (2 or more convulsions in 24 h), impaired consciousness (Blantyre coma score < 3), jaundice [plasma bilirubin > 50 µmol/L (> 3 mg/dL)], pulmonary oedema/respiratory distress (Kussmaul’s breathing manifesting as deep breathing with signs of increased work of breathing) and haemoglobinuria, hyperparasitaemia (greater than 10% in highly endemic areas), acidosis (venous plasma lactate > 5 mmol/L), hypoglycaemia (< 2.2 mmol/L or < 40 mg/dL), lactic acidosis (lactate > 5 mol/L), renal failure (serum creatinine > 3 mg/dL/BUN > 20 mmol/L). Malaria diagnosis in this facility was done using blood smear and rapid diagnostic test kits, both of which can identify the *P. falciparum* species. Children with no clear diagnosis and children admitted with other diagnosis other than malaria were excluded from the study.

### Data collection and management

Using a customized proforma, data were abstracted on biological and social demographic characteristics such as age and gender. The study collected clinical features of severe malaria according to the WHO 2015 clinical surveillance criteria for SM described above and as previously applied in Eastern Uganda [13, 14]. In addition, outcome data on length of hospital stay considered as normal if ≤ 4 days and prolonged if > 4 days (classified basing on the 75th percentile length of hospital stay) was collected. Furthermore, data on survival were captured as either alive or dead. In-patient admission registers were used to obtain records of total admission and the diagnosis at discharge was considered as the final cause of admission. Patient level data were reviewed and abstracted from the paediatric admission records. The WHO guidelines were used to classify SM.

### Data analysis

Data were coded and entered in excel where it was cleaned and exported to STATA statistical package for further management and analysis. The data were analysed at three different levels of univariate, bivariate and multivariate. At univariate level, descriptive statistics were summarized as frequency, proportions, mean and median. Binary logistic regression was done to determine the association between independent and outcome (Length of Hospital stay) variables, while Fishers’ exact test was used to determine risks associated with death at bivariate level. Multiple logistic regression was used to determine the factors associated with prolonged hospital stay at multivariate level.

## Results

### Demographic and clinical characteristics of patients admitted with severe malaria

**Fig. 1:**
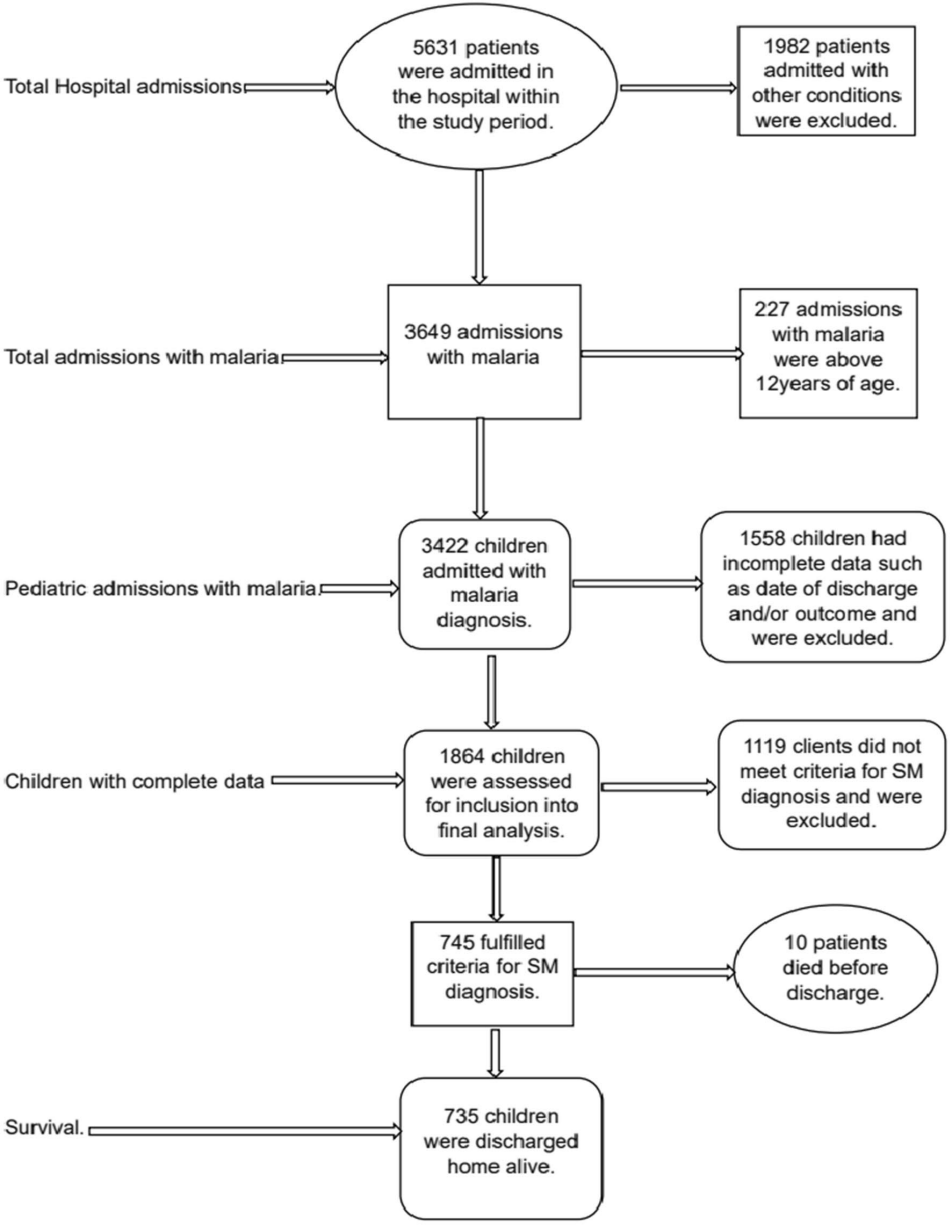
Flow chart showing participant enrolment into the study

A total of 5631 admission records were retrieved for this study period. Of these 3649 (64.8%) were malaria admissions and 3422/3649 were children below 12 years. There was high level of data incompleteness as 1558/3422 (45.5%) children admitted had incomplete data as date of discharge and status at discharge and were excluded from further assessment and analysis. Of the 1864 children who had complete data and were assessed for eligibility of diagnosis of severe malaria, 745 (40.0%) fulfilled the severe malaria inclusion criteria (Fig. 1).

### Clinical presentation of severe malaria

**Fig. 2:**
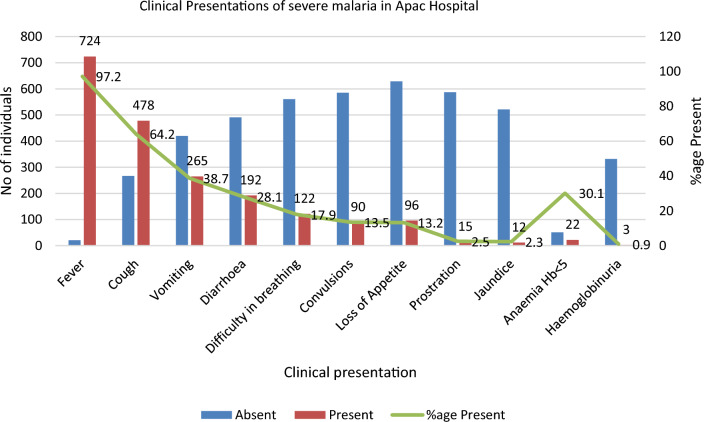
Clinical presentation of severe malaria in children under 12 years in Apac District Hospital

The most common clinical presentations among these children with severe malaria in Apac District Hospital included; (a) fever 722 (97.3%), (b) cough 478 (64.2%), (c) vomiting 265 (38.7%), (d) diarrhea 192 (28.1%) and (e) difficulty in breathing122 (17.9%), anemia 22 (30.1% among those tested) and hemoglobinuria 3 (0.9% among those tested) (Fig. 2).

Of the 745 children, 51.4% (n = 381) were males. The median age at admission was 31 months (IQR = 17–60). Survival with SM in Apac District Hospital is high, as death rate is low at 1.3%. Despite the low numbers, the factors that had statistical association (Fisher’s exact) with death were fever (*P =* 0.030), and haemoglobinuria (*P =* 0.027) (Table 1).

**Table 1:** Survival outcomes of SM in Apac District Hospital

Variable	Overall, N (%)	Survivors, N (%)	Death, N (%)	P-value
Number	745	735	10	
Age/month				
Median (IQR)	31 (17–60)	31 (17–60)	67 (26–108)	0.541
Gender				
Male	381/745 (51.4)	375/735 (51.0)	6/10 (60.0)	0.753
Clinical symptoms				
Fever	724/745 (97.2)	716/735 (97.4)	8/10 (80.0)	**0.030**
Cough	478/745 (64.2)	473/735 (64.4)	5/10 (50.0)	0.342
Vomiting	265/685 (38.7)	261/677 (38.6)	4/8 (50.0)	0.494
Diarrhoea	192/683 (28.1)	190/675 (28.2)	2/8 (25.0)	1.00
Loss of appetite	96/725 (13.2)	93/716 (13.0)	3/9 (33.3)	0.109
Convulsions	91/676 (13.5)	90/668 (13.5)	1/8 (12.5%)	1.00
Haemoglobinuria	3/335 (0.9)	2/332 (0.6)	1/3 (33.3)	**0.027**
Respiratory symptoms				
Difficulty in breathing	122/683 (17.9)	121/675 (17.9)	1/8 (12.5)	1.00
Hypoxia (O_2_ Sat < 90%)	6/7 (85.7)	4/5 (80.0)	2/2 (100.0)	1.00
Clinical signs				
General				
Pallor	16/531 (3.0)	15/525 (2.9)	1/6 (16.8)	0.168
Jaundice	12/533 (2.3)	11/526 (2.1)	1/7 (14.3)	0.148
Cardiovascular/hydration				
Sunken eyes	6/527 (1.1)	5/521 (1.0)	1/6 (16.7)	0.067
Neurological				
Impaired consciousness	9/605 (1.5)	8/598 (1.3)	1/7 (14.3)	0.100
Prostration	15/602 (2.5)	14/595 (2.4)	1/7 (14.3)	0.163
Laboratory				
HB < 5 g/dL	22/73 (30.1)	20/66 (30.3)	2/7 (28.6)	1.00
Hyper-parasitaemia, BS + 3 Mps	31/82 (37.8)	31/82 (37.8)	0/0	

Bold = significant with P < 0.05, Fisher’s exact test

The mean length of hospital stay was 3.3 (± 3.0) days, while the median length was 2 (IQR; 2–4) days and 133 (17.9%) of the study participants stayed in the hospital for more than 4 days (Prolonged stay). At multivariate level, factors that were significantly associated with prolonged hospital stay were difficulty in breathing, aOR 1.83 (95% CI 1.02–3.27, *P =* 0.042) compared to those with normal breathing, and prostration aOR 8.47 (95% CI 1.94–36.99, *P =* 0.004) compared to those without prostration (Table 2).

**Table 2:** Factors associated with prolonged hospital stay among severe malaria children at Apac District Hospital

Variable	Overall, N	Length of hospital stay		Crude OR (95% CI)	Adjusted OR (95% CI)	*P*-value
	N = 745	Normal stay, n (%), n = 612	Prolonged stay, n (%), n = 133			
Age/month						
Median (IQR)	31 (17–60)	31 (18–60)	28 (12–72)	1.00 (1.00–1.01)	1.00 (1.00–1.01)	0.621
Gender						
Male	381/745 (51.1)	326/616 (53.3)	55/133 (41.4)	**0.62 (0.42–0.90)**	0.66 (0.41–1.07)	0.094
Clinical symptoms and signs						
Fever	724/745 (97.2)	596/612 (97.4)	128/133 (96.2)	0.99 (0.25–1.91)		
Cough	478/745 (64.2)	393/612 (64.2)	85/133 (63.9)	0.99 (0.67–1.46)		
Vomiting	265/685 (38.7)	221/563 (39.3)	44/122 (36.1)	0.87 (0.58–1.31)		
Diarrhoea	192/683 (28.1)	157/559 (28.1)	35 (28.2)	1.01 (0.65–1.55)		
Loss of appetite	96/725 (13.4)	69/594 (11.6)	27/131 (20.6)	**1.98 (1.21–3.23**)	1.51 (0.77–2.94)	0.227
Convulsions	91/676 (13.5)	72/555 (13.0)	19/121 (15.7)	1.25 (0.72–2.16)		
Jaundice	12/533 (2.3)	5/433 (1.2)	7/100 (7.0)	**6.44 (2.00-20.75)**	1.48 (0.22–9.75)	0.685
Difficulty in breathing	122/683 (17.9)	92/561 (16.4)	30/122 (24.6)	**1.66 (1.04–2.66)**	**1.83 (1.02–3.27)**	**0.042**
Pallor	16/531 (3.0)	7/430 (1.6)	9/101 (8.9)	**5.91 (2.15–16.28)**	3.87 (0.78–19.22)	0.097
Prostration	15/602 (2.5)	8/494 (1.6)	7/108 (6.5)	**4.21 (1.49–11.87)**	**8.47 (1.94–36.99)**	**0.004**

Bold = significant with P < 0.05. All factors were adjusted for each other

## Discussion

This study sought to determine the burden and clinical spectrum of severe malaria from routine health facility-based surveillance at Apac district hospital. The median age at admission of 31 months (IQR = 17–60) observed in this study is a younger median age compared to the current trend of data reporting increasing age with malaria epidemiological transition [14]. This also indicates that malaria epidemiological transition is heterogenous across different settings with areas experiencing high transmission like Apac district showing younger age remains highly vulnerable to SM. The most common clinical presentations among children with SM in this study were non-specific and consistent with most reported data on SM in SSA [15–18]. Moreover, the common findings which were consistent with the WHO surveillance criteria included respiratory distress (17.9%), convulsions (13.5%), impaired consciousness (1.5%), anaemia (30.1%), and haemoglobinuria (0.9%). These findings are similar to studies undertaken in India, Ghana and Cameroon [15–18]. This study reports a high prevalence of severe anaemia (30.1%) compared to data in Eastern and Central Uganda, but this could have been because of a selected group of patients since only 73/745 (9.8%) had haemoglobin estimation on admission. Even then it compares well with other larger studies in Africa and Brazil, where severe anaemia was reported as the commonest manifestation of SM in younger children [19–21]. Besides, other studies have reported even higher prevalence of severe anaemia in SM at 61.4% [9], 55% [17], 37% [22] and 32% [18].

Some data have identified factors affecting length of hospital stay after admission with malaria as sex (females), age (younger patients), delay seeking treatment, acute renal failure and third line treatment [23]. In this study, the median length of hospital stay was 2 (IQR; 2–4) days, but a higher proportion 133 (17.9%) had prolonged hospital stay (> 4 days). Compared to other studies [23], this study reports difficulty in breathing, aOR 1.83 (95% CI 1.02–3.27, *P =* 0.042) and prostration aOR 8.47 (95% CI 1.94–36.99, *P =* 0.004) to be associated with prolonged hospital stay. Elsewhere, both of these factors have historically been reported to be associated with mortality outcomes [24]. Mortality in this study was at 10 (1.3%) and sets a new record since most studies have previously reported death rate of 3% and above [15, 21, 22] and the WHO estimates mortality due to SM at 10–20% [13]. This could possibly be due to several factors. Firstly, use of artesunate which is WHO-recommended first-line treatment for SM [20]. Secondly, Apac district has been cited to have the highest malaria transmission ever reported in the world [25], enlisting a potential role of persistent partial immunity following repeated exposure modifying disease outcomes but this needs to be established in further research. In the Central part of Uganda, at the Mulago national referral hospital, mortality due to SM was reported at 6.0%, with cerebral malaria and lactic acidosis accounting for most of the deaths [9]. Elsewhere, a randomized clinical trial in nine African countries with 11 study sites reported death resulting from SM at 9.7% [20]. Another study in Ghana reported a mortality of 11.2% [17]. Other studies found with high incidences of death include a study on determinants of fatal outcomes of severe malaria in children in Cotonou-Benin that reported a mortality of 13.1% mortality [26]. Furthermore, a study on SM and risk factors for mortality in the Democratic Republic of Congo that reported even higher rates of 28.3% [27]. This study reports haemoglobinuria as risk factors for death, but is not frequently reported in other series outside of studies in Eastern Uganda [14, 28]. The commonly reported predictors of mortality in SM include coma and convulsions [20, 26, 27], malnutrition [17, 27], cerebral malaria [21, 22], and hypoglycaemia [17].

There were limitations that may have affected the study finding. The SM assessments were mainly clinical since laboratory tests were limited. For instance, no tests were reported for serum electrolytes, blood sugar levels and renal function tests. In addition, the clinical data were incomplete in most of the variables except for fever which seems to have been voluntarily reported by caregivers. Very small samples were observed among death, and this made it difficult to reliably determine the predictors of death. This study, however, has been able to report that a high proportion (20.4%) of malaria admissions were due to SM. In addition, a description of SM within the limits of these data has been done. In Apac and most settings in Uganda, just as it is in many parts of SSA, active surveillance of SM is not only costly but also labour-intensive and, therefore, not sustainable. In these settings, routinely collected data represent untapped resource as primary surveillance data that also may complement research data when available. Priority to use routinely collected data needs to be directed to harnessing their potential to contribute to the burden, descriptions, and clinical spectrum of SM. For a complete picture on the burden and disease spectrum, emphasis on high quality clinical and laboratory data are critical.

## Conclusion

This study was able to use routinely collected data to describe the burden and clinical spectrum of SM. Prolonged hospital stay was associated with prostration and difficulty in breathing. This study has reported a new record low mortality of 1.3% in malaria endemic and high transmission settings. Improvement in the quality of data from such settings would improve disease descriptions for policy, monitoring of response to interventions and to inform research.

## Data Availability

The study data are available by request to the corresponding author.
